# Osteosarcoma pulmonary metastasis: the hemodynamic mechanical-filtration hypothesis for subpleural predominance

**DOI:** 10.3389/fonc.2026.1763065

**Published:** 2026-04-14

**Authors:** Kaihua Zhang

**Affiliations:** 1 Department of Thoracic Surgery, Beijing Genertec Aerospace Hospital, Beijing, China; 2 Department of Thoracic Surgery, The First Hospital of Tsinghua University, Beijing, China

**Keywords:** biomechanical metastasis, circulating tumor cell clusters, hemodynamic filtration, osteosarcoma, pulmonary metastasis, pulmonary microcirculation, subpleural predominance

## Abstract

**Background:**

Pulmonary metastasis remains the leading cause of death in osteosarcoma; however, the pronounced and long-recognized subpleural predominance of metastatic nodules remains mechanistically unexplained. Contemporary “seed-and-soil” and pre-metastatic niche frameworks emphasize microenvironmental conditioning and spatial heterogeneity, but they do not explain why metastatic seeding consistently localizes to the peripheral 1–2 cm of the lung. Likewise, modern mechanical perspectives acknowledge that circulating tumor cells can be retained at multiple sites within the pulmonary microvasculature (including capillaries and pre-capillary arterioles), yet they do not account for a reproducible intra-organ, subpleural spatial bias.

**Knowledge gap:**

A testable model that links pulmonary microvascular geometry and hemodynamics to the consistent subpleural localization of early osteosarcoma metastatic seeding has yet to be established.

**Hypothesis:**

We propose the Hemodynamic Mechanical-Filtration Hypothesis: a clinically relevant subset of osteosarcoma circulating material may travel as larger, relatively stiff embolic units, including circulating tumor cell clusters (CTC clusters) and/or complex aggregates involving platelets and fibrin (CTC–platelet aggregates). Because these units have less instantaneous deformability than single cells, they may undergo size-selective mechanical arrest in terminal pre-alveolar arterioles (20–40 μm), which constitute a functional bottleneck immediately proximal to the capillary bed. We further hypothesize that filtration-prone distal arteriolar segments are preferentially distributed within a defined subpleural distance, an explicitly testable quantitative anatomical premise. This initial mechanical arrest establishes a spatial template for seeding, upon which post-arrest metastatic niche maturation and outgrowth can proceed, potentially modulated by upstream pre-metastatic niche (PMN) priming.

**Approach:**

The hypothesis integrates pulmonary microvascular anatomy, tumor biomechanics, cluster/aggregate rheology, and hemodynamic gradients. We outline falsifiable predictions and multi-level validation strategies, including intravital imaging, compliant/endothelialized microfluidic models, computational flow simulations, and clinical spatial mapping of metastatic nodules as a function of distance from the pleura.

**Key insights:**

By explicitly coupling embolic physical properties with hierarchical vascular geometry and regional hemodynamics, this framework provides a spatially resolved extension of mechanical arrest models and generates testable explanations for subpleural predominance as well as for the conditions under which the site of arrest may shift.

**Significance:**

If validated, this hypothesis would reframe early osteosarcoma lung seeding as a process constrained by microvascular biomechanics and could guide future mechanistic studies aimed at developing preventive strategies targeting embolic integrity and pulmonary microcirculatory conditions.

## 1 Introduction

Osteosarcoma (OS) represents a significant clinical challenge in pediatric and young adult oncology, with pulmonary metastasis being the leading cause of death (2). Two foundational concepts have historically dominated metastatic theory: Paget’s “Seed and Soil” hypothesis (1889), which emphasizes the compatibility between the metastatic cell (“seed”) and the organ microenvironment (“soil”) (3), and Ewing’s “Circulatory Mechanics” theory (1928), which attributes metastasis patterns largely to passive transport and mechanical arrest in the first encountered capillary bed (4).

Contemporary metastasis biology has expanded Paget’s framework by recognizing that the “soil” is spatially and cellularly heterogeneous within organs, and that metastatic success depends on localized biochemical, mechanical, and immunological cues rather than whole-organ averages (5). Circulating tumor cell clusters and platelet-associated aggregates have been increasingly recognized as important mediators of metastatic dissemination (1). In parallel, modern mechanobiology has refined mechanical arrest concepts beyond a strict “capillary-only” interpretation, acknowledging that retention may occur across multiple microvascular levels depending on tumor cell size, deformability, hemodynamics, and vascular interactions.

Within this evolving landscape, the PMN concept has provided a molecular extension of “Seed and Soil”: primary tumor–derived systemic factors can condition distant organs and prime permissive microenvironments prior to tumor cell arrival (6). While such frameworks help explain OS tropism for the lung (7), a key mechanistic gap remains: why metastatic nodules reproducibly concentrate in the subpleural lung rather than distributing more uniformly across central and peripheral parenchyma. Addressing this intra-organ spatial specificity requires integrating molecular priming concepts with spatially resolved vascular anatomy and hemodynamic constraints.

## 2 Clinical observation and the need for a refined hypothesis

### 2.1 Clinical observation

**Figure 1:**
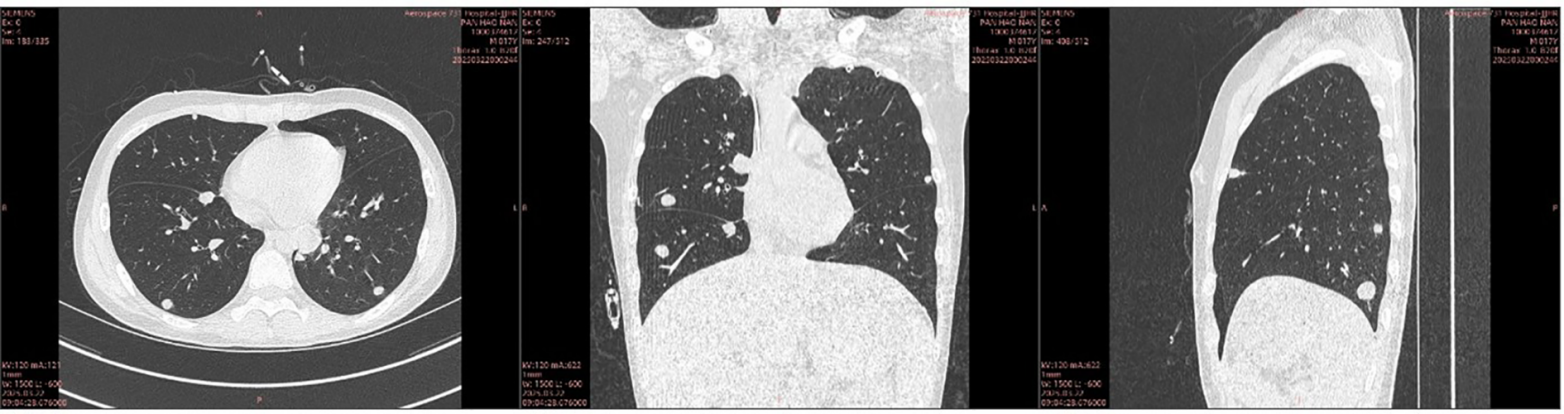
Multimodal computed tomography (CT) images illustrating the consistent subpleural predominance of pulmonary metastases in a 17-year-old male osteosarcoma patient.

Although PMN-related molecular and immunological processes are often discussed at the organ-wide scale, OS pulmonary metastases exhibit a consistently subpleural predominance on clinical imaging (CT, PET) (8). In a recent quantitative retrospective series of patients undergoing pulmonary metastasectomy, more than 80% of resected nodules were located subpleurally, frequently amenable to minimally invasive wedge resection (9). Similar descriptions appear in classic surgical and oncology texts. This disparity, in which peripheral lesions substantially outnumber central lesions, highlights the need for a mechanistic explanation of intra-organ spatial specificity in OS lung metastasis (**Figure 1**).

### 2.2 The need for a refined hypothesis (the disconnect: limitations of existing theories)

The subpleural bias, whereby metastases cluster near the visceral pleura while central lung fields are relatively spared, remains difficult to reconcile with prevailing explanatory frameworks for explaining within-lung spatial patterning.

#### 2.2.1 PMN/molecular models

Modern PMN and “Seed and Soil” models explicitly recognize microenvironmental heterogeneity and provide compelling mechanisms for organotropism. However, in osteosarcoma, these models have not yet articulated a concrete, quantitative mechanism explaining why the peripheral 1–2 cm of lung parenchyma would be consistently and disproportionately permissive for initial seeding compared with more central regions (5). In particular, a spatially reproducible pattern suggests that, beyond molecular priming, anatomical and physical constraints may contribute an additional “first-pass” determinant of where seeding occurs (7).

#### 2.2.2 Mechanical arrest models (from classical to contemporary)

Mechanical perspectives have traditionally emphasized size-restricted trapping in narrow microvessels, and contemporary work further indicates that arrest can occur not only in capillaries but also in pre-capillary arterioles depending on cell/cluster size, deformability, hemodynamics, and vascular interactions. Yet, even within this modern view, a specific mechanism that produces a robust subpleural spatial bias is still missing. If osteosarcoma circulating entities were predominantly small and highly deformable and were retained mainly within the widely distributed capillary bed (diameter ~5–8 μm) (10), one would expect a more diffuse intralobular distribution rather than dense peripheral clustering. This motivates a refined hypothesis in which the effective size/rigidity of disseminating embolic units and the hierarchical geometry of distal pulmonary arterioles together impose a spatially biased filtration process that preferentially seeds the subpleural lung.

The clinical reality demands a unifying hypothesis that integrates the physical properties of the “seed” with the biomechanical constraints of the “path.” We propose that subpleural predominance is driven not by a unique PMN signature, but by a biomechanical filtration process rooted in vascular anatomy, representing a microcirculatory refinement of Ewing’s circulatory mechanics.

## 3 The hemodynamic mechanical-filtration hypothesis

We propose that the subpleural predominance of OS pulmonary metastases arises from a size-selective mechanical filtration mechanism operating at distal, pre-capillary segments of the pulmonary arterial tree. In this framework, embolus physical properties (effective size and deformability) interact with hierarchical vascular geometry and regional hemodynamics to bias where early arrest occurs. Importantly, this hypothesis is intended to be complementary to intra-organ microenvironmental heterogeneity (i.e., subpleural “soil” differences), rather than excluding it.

Specifically, we hypothesize that a clinically relevant subset of disseminating OS material may circulate as larger and mechanically less deformable embolic units, including CTC clusters and/or CTC–platelet aggregates (11). Such units may preferentially undergo mechanical arrest in terminal pre-alveolar arterioles (20–40 μm), a functional bottleneck just upstream of the capillary bed. In the gas-exchanging region, small intra-acinar pulmonary arteries with inner diameters on the order of ~20–40 μm, located near the alveolar septal “gussets,” can be regarded as terminal pre-alveolar arteriolar segments functionally relevant for size-based filtration. We further propose that filtration-prone distal arteriolar segments are enriched within a defined subpleural zone, an explicit quantitative anatomical premise that requires direct mapping and validation.

This hypothesis posits a sequential two-stage process:

Stage I: Physical arrest (modern Ewing: spatially refined).

Large, relatively rigid embolic units experience size-selective mechanical arrest at distal pre-capillary arteriolar bottlenecks, biasing initial seeding toward the lung periphery and subpleural zone.

Stage II: Post-arrest adaptation (metastatic niche maturation).

Following arrest (and subsequent extravasation where applicable), lodged OS cells interact with local endothelial cells, stromal elements, immune cells, and extracellular matrix to remodel the surrounding microenvironment in ways that promote survival and outgrowth, referred to here as metastatic niche maturation. PMN priming, if present, may act as an upstream permissive background but is not initiated by mechanical arrest.

Together, this framework provides a spatially resolved extension of contemporary mechanical arrest models by specifying (i) the embolic physical state of the “seed” and (ii) the distal microvascular segments most likely to function as a size-selective filter that can generate a reproducible subpleural spatial bias.

## 4 Mechanistic rationale

The proposed model relies on the specific interplay between tumor cell biomechanics, the hierarchical structure of the pulmonary vasculature, and fluid dynamics.

### 4.1 The biomechanics of OS circulating material: a rigid and clustered seed

The physical state of circulating tumor material is a critical determinant in any mechanical-filtration model. CTCs can exist as single cells, multicellular clusters, and CTC–platelet aggregates (12). Platelets may further enhance this process by rapidly coating circulating tumor cells, promoting heterotypic platelet–CTC aggregation and fibrin-rich microthrombus formation. These composite embolic units are likely to have greater effective size and reduced instantaneous deformability, which may increase their propensity for stable mechanical trapping in distal pulmonary arterioles and thereby contribute to the observed subpleural bias (1, 13, 14). For many epithelial tumors, single CTCs (typically ~10–20 μm) can deform sufficiently to traverse capillaries, and arrest may rely more heavily on adhesion-mediated mechanisms (15). In osteosarcoma, we propose that a clinically meaningful subset of circulating entities may be larger and mechanically less deformable, thereby increasing the likelihood of size-dependent microvascular retention.

Cluster formation: plausible, but not exclusive. Considering the inefficiency of tumor metastasis and the fact that most CTCs die within a short period (16), it is currently believed that multicellular clusters, as well as CTC–platelet aggregates, are more likely to mediate successful metastatic dissemination (17). Although direct evidence in osteosarcoma is limited, multicellular CTC clusters, which have been widely documented in various carcinomas and shown to harbor significantly higher metastatic potential than single CTCs, represent one of the most plausible mechanisms for osteosarcoma dissemination.

Beyond serving as passive coating elements, platelets may actively reshape the biomechanical behavior of circulating osteosarcoma material. Through rapid adhesion to tumor cells, platelet activation can facilitate the formation of heterotypic CTC–platelet aggregates, thereby increasing aggregate cohesion, effective diameter, and resistance to hemodynamic shear. In addition, platelet-rich embolic units may interact more efficiently with the pulmonary endothelium and remain lodged once captured at distal narrowing segments. Within the framework of our hypothesis, these effects would not replace geometric filtration, but would amplify it by converting otherwise deformable circulating material into more embolus-like units that are mechanically predisposed to arrest in terminal pre-capillary arterioles (14, 18).

Intrinsic rigidity (contextualized and testable): Unlike pliable epithelial CTCs, OS cells produce a rich osteoid extracellular matrix (tumor osteoid), a pathognomonic hallmark of the disease (19), which results in a dense, stiff tumor stroma that likely imposes substantial mechanical constraints on both tumor cells and their microenvironment (20). When they cluster, this inherent rigidity, amplified by the addition of platelets and fibrin in thrombi, significantly reduces the collective deformability of the aggregate. These structures act effectively as solid embolic units, making their passage through narrow vessels strictly dependent on the vessel’s minimum diameter, not only on biological adhesion or cellular squeezing.

**Figure 2:**
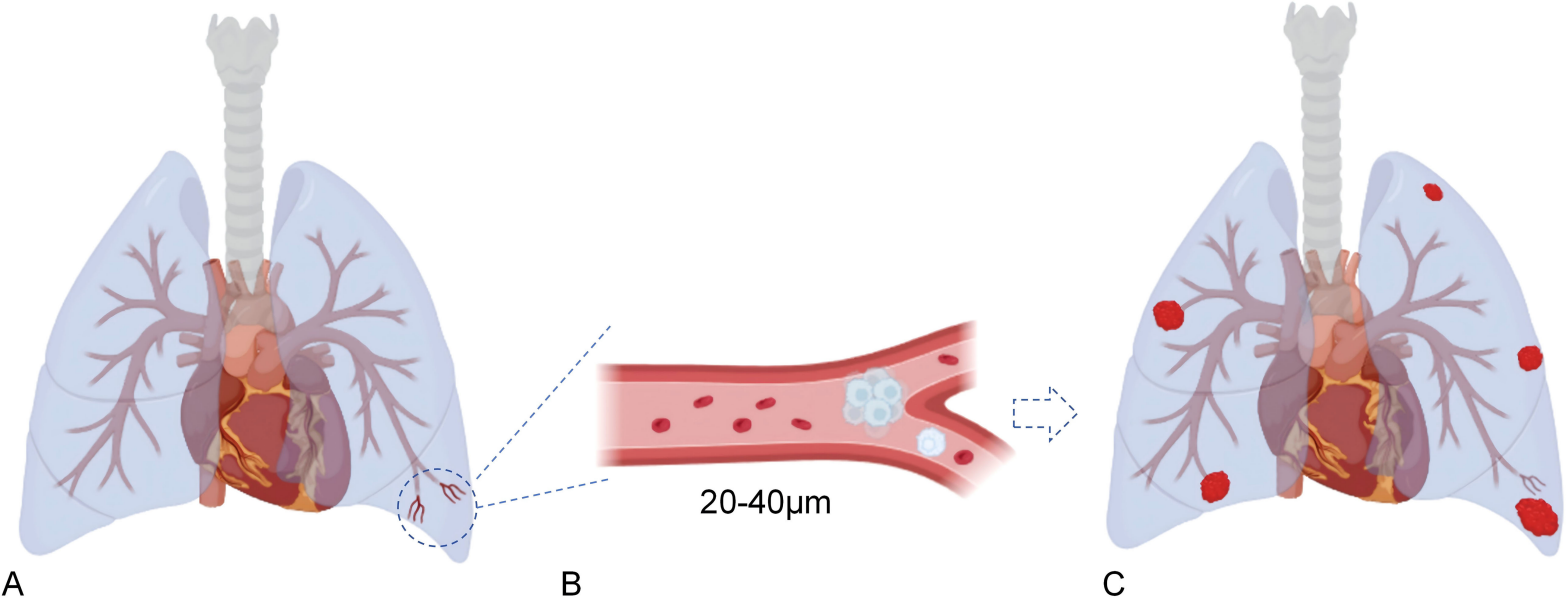
Size-selective mechanical filtration of osteosarcoma CTC clusters in terminal pulmonary arterioles. **(A)** Schematic overview of the lung showing the peripheral subpleural region where terminal pre-alveolar arterioles are anatomically enriched. These distal vessels represent the final diameter-restrictive branching points within the pulmonary arterial tree before blood enters the capillary network. **(B)** Magnified depiction of a terminal arteriole (20–40 μm) demonstrating size-selective mechanical arrest. Rigid, multicellular osteosarcoma CTC clusters or CTC–platelet aggregates fail to traverse the narrowing vessel due to limited deformability, while erythrocytes pass freely. This physical bottleneck determines the initial spatial site of metastatic seeding. **(C)** Resulting metastatic pattern within the lung, illustrating the preferential accumulation of metastatic nodules in the subpleural region. This distribution reflects the underlying biomechanical filtration mechanism imposed by microvascular geometry and hemodynamic flow conditions.

This distinction is crucial: if an OS cluster is 30 μm in diameter, it cannot pass through a 25 μm vessel, regardless of molecular signaling. This physical constraint is the basis of the mechanical filter (**Figure 2**).

### 4.2 The pulmonary arterial tree: a hierarchical size-selective filter

The lung vasculature is not a uniform mesh but a highly organized hierarchical structure, creating predictable gradients in diameter, pressure, and shear stress.

#### 4.2.1 Anatomical graduation and the locus of arrest

The pulmonary circulation begins with the large pulmonary arteries (millimeters in diameter) and branches successively, creating a progressive reduction in vessel caliber.

Taken together, **Table 1** and **Table 2** suggest that terminal pre-alveolar arteriolar segments are plausible anatomical candidates for size-selective filtration of larger, less deformable embolic units. These vessels represent the final diameter-restrictive branching points before blood enters the capillary network. While the capillary bed can retain small and deformable entities under certain conditions, distal arteriolar bottlenecks may constitute the first obligate geometric barrier encountered by larger embolic units after they have traversed wider central vessels.

**Table 1:** Physical fate mapping of OS circulating entities: size-based arrest potential.

Circulating tumor entity	Typical size (diameter, *µ*m)	Primary physical property	Main arrest site in pulmonary vasculature	Arrest potential
Single-Cell CTC	10-20	High Deformability	Pulmonary Capillary Bed (5-8 *µ*m)	Low (Prone to washout)
CTC Cluster / Small Aggregate	20-40	High Rigidity, Low Deformability	Terminal Pre- alveolar Arteriole	Highest (Critical Size Match)
Large CTC- Fibrin Thrombus	> 40 (Up to 100+)	Extreme Rigidity, Solid Embolic Behavior	Proximal Pre- capillary Arteriole (Upstream)	Very High (Early embolism)

**Table 2:** Hierarchical vascular filtration: anatomical role of pre-capillary vessels in spatial arrest.

Vascular structure	Diameter range (*µ*m)	Filtering role	Spatial significance in OS metastasis
Pulmonary Capillary	5-8	Final filter for single cells (Molecular adhesion)	Uniformly distributed throughout lung parenchyma
Terminal Pre- alveolar Arteriole	20-40	Primary filter for OS clusters (Mechanical Sizing)	Subpleural enrichment (Focus of Hypothesis)
Pre-capillary Arteriole (General)	20-100+	General filtration of large emboli	Distributed throughout parenchyma (Less concentrated peripherally)

#### 4.2.2 The subpleural representation of distal filtration segments

Based on established pulmonary vascular anatomy, the lung periphery (including the subpleural zone) contains distal arteriolar branches and capillary beds that supply the outermost lobular/acinar units (21). We hypothesize that distal, filtration-relevant arteriolar segments may be preferentially represented within a defined subpleural distance, thereby biasing the spatial location of size-based mechanical arrest toward the pleural surface. However, the degree of such subpleural representation is a quantitative anatomical question that requires direct validation (e.g., vascular casting and micro-CT mapping, or histologic distance-to-pleura analyses of arteriolar density). If confirmed, this spatial vascular geometry would provide a parsimonious anatomical substrate for reproducible subpleural seeding.

### 4.3 Hemodynamic gradients: conditions favoring stable lodging

Beyond geometry, pulmonary hemodynamics may modulate the probability that a mechanically retained embolic unit remains stably lodged rather than fragmenting or being dislodged (18).

#### 4.3.1 Decreasing shear stress

Blood flow velocity and wall shear stress (WSS) generally decline from central pulmonary arteries toward more distal branches, as suggested by image-based computational fluid dynamics studies of the pulmonary vasculature (22, 23). Higher shear environments may promote fragmentation of loosely cohesive aggregates and reduce the stability of lodging, whereas lower shear conditions in distal arterioles may permit more persistent mechanical retention once an embolic unit is geometrically constrained.

#### 4.3.2 Flow separation and bifurcations

Near bifurcations, complex velocity and shear profiles can promote margination of circulating particles and clusters toward vessel walls or branch points (24), in smaller distal vessels, reduced turbulence and lower inertial forces may further favor stable lodging of a captured embolic unit at a narrowing segment, thereby increasing the opportunity for endothelial interaction, extravasation, and subsequent metastatic niche maturation.

Taken together, these considerations suggest that the subpleural region may provide a hemodynamically permissive setting for stable mechanical arrest when distal vessel diameters overlap with the effective size and deformability spectrum of circulating OS embolic units. This inference remains experimentally testable.

This effect may be especially relevant for platelet-coated CTC clusters or fibrin-rich microthrombi, whose increased cohesion and altered surface interactions could favor persistent lodging under low-shear distal flow conditions.

## 5 Testable predictions and falsifiability

The Hemodynamic Mechanical-Filtration Hypothesis generates a set of falsifiable predictions that distinguish it from (i) purely organ-wide PMN explanations and (ii) non–spatially resolved “capillary-only” mechanical arrest models. Importantly, these predictions separate PMN priming (pre-arrival conditioning) from post-arrest metastatic niche maturation (local remodeling after arrest/extravasation), which are temporally and mechanistically distinct processes.

Prediction 1: embolus-size–dependent spatial bias.

If larger, less deformable embolic units (clusters/aggregates) drive peripheral seeding, then the subpleural bias should scale with the effective diameter/deformability distribution of circulating OS entities.

Falsification: no association between embolus size/deformability and pleural distance of early foci.

Prediction 2: deformability manipulation shifts arrest location.

Interventions that increase deformability or reduce cluster integrity (pharmacologic/genetic) should shift initial arrest away from distal 20–40 μm bottlenecks toward more distal capillary-level arrest and/or reduce stable lodging.

Falsification: robustly verified changes in cluster mechanics do not alter the spatial pattern of early arrest or later metastasis distribution.

Prediction 3: mechanical arrest precedes (and does not initiate) PMN priming signals.

Early signatures of physical retention or obstruction (e.g., microvascular arrest at distal arterioles) should be detectable at the earliest time points, whereas PMN-priming markers, if present, should precede tumor-cell arrival rather than arise as a consequence of arrest.

Falsification: evidence that PMN priming is absent pre-arrival yet appears only after arrest as a defining PMN feature (i.e., inability to distinguish PMN from post-arrest niche maturation).

Prediction 4: post-arrest niche maturation is spatially localized around arrested emboli.

After arrest, local endothelial/stromal/immune remodeling consistent with metastatic niche maturation should occur preferentially around the arrested embolus and predict outgrowth probability. This remodeling may occur on top of, and be facilitated by, any pre-existing PMN priming.

Falsification: microenvironmental remodeling is uniformly distributed and unrelated to the location of arrested emboli.

Prediction 5: hemodynamic modulation changes capture efficiency and/or stability.

Experimental modulation of distal arteriolar caliber and hemodynamic conditions should alter embolic capture, lodging stability, fragmentation, and displacement, particularly for emboli near the critical size threshold.

Falsification: spatial arrest patterns remain unchanged despite verified hemodynamic/diameter modulation in the distal microcirculation.

Prediction 6: anatomical correspondence between earliest foci and distal arteriolar structures.

The earliest identifiable microscopic OS foci should exhibit preferential proximity to distal pre-capillary arteriolar structures compared with random parenchymal locations, consistent with an arteriolar filtration locus.

Falsification: early foci show no preferential association with distal arterioles (e.g., random location or capillary-only association).

Prediction 7: cross-tumor generalizability based on embolic biomechanics.

Tumors that more frequently disseminate as cohesive clusters/aggregates with higher effective stiffness should show stronger subpleural (or distal) bias than tumors disseminating mainly as small, highly deformable single cells.

Falsification: No relationship between a tumor’s circulating biomechanical profile and intra-lung metastatic spatial distribution across tumor types.

## 6 Detailed experimental designs to test the hypothesis

The hypothesis can be evaluated using a three-tiered strategy: (i) *in vivo* models to map early arrest locations and subsequent outgrowth, (ii) controlled *in vitro/ex vivo* platforms to isolate biomechanical filtration mechanisms under defined flow conditions, and (iii) clinical imaging and pathology studies to quantify spatial patterns in humans. Across all tiers, experimental readouts should separately capture (a) the physical arrest step and (b) post-arrest metastatic niche maturation, while allowing PMN priming to be assessed as an upstream modifier rather than a post-arrest event.

### 6.1 *In Vivo* animal models: tracking the arrest

#### 6.1.1 Differential dissemination and mapping

Protocol: Use an immunocompetent murine model (e.g., syngeneic OS) and label OS cells with distinct fluorophores (e.g., Red for single cells, Green for clusters/thrombi). Inject three groups via the tail vein: 1) Isolated single CTCs (prepared via mechanical sieving/FACS), 2) Native CTC clusters, and 3) CTC–platelet aggregates (prepared by co-incubating clusters with thrombin/fibrinogen).

Readout: Sacrifice animals at very early time points (1h, 6h, 24h). Image the intact lung using fluorescent stereo microscopy and micro-CT. Use 3D spatial mapping software to calculate the distance of each arrested embolus from the nearest pleural surface.

Expected support: The “cluster” and “thrombi” groups will exhibit a highly non-random, significantly shorter mean distance from the pleura compared to the “single CTC” group, which should show a more uniform central/peripheral distribution.

#### 6.1.2 Pharmacologic modulation of cluster integrity

Protocol: Establish a stable OS model. Treat one group with agents known to disrupt the stability of tumor cell clusters or associated fibrin (e.g., low-dose fibrinolytic agents or anti-platelet therapy) 1 hour prior to and 1 hour post-injection. The control group receives vehicle.

Readout: Quantify the size of circulating tumor aggregates in both groups pre- and post-treatment. After 3–4 weeks, quantify and map the spatial location (subpleural *vs*. central) of resulting macroscopic metastases via CT.

Expected support: A confirmed reduction in circulating cluster size should lead to a statistically significant reduction in the number of subpleurally located metastases and an increase in centrally located ones, or a reduction in overall metastasis burden due to less successful seeding.

### 6.2 *Ex vivo* and *in vitro* biomechanical models: controlled filtration

#### 6.2.1 Microfluidic vascular mimics

Protocol: Fabricate microfluidic chips containing two distinct filtration zones: 1) a central channel that branches into vessels of 80–100µm, and 2) a distal zone that branches into parallel terminal channels of 20–40µm (simulating pre-alveolar arterioles) before converging into 5-8 µm post-arteriolar channels. Perfuse OS single cells and clusters at physiological pulmonary flow rates and pressures.

Readout: Use high-speed microscopy to track the trajectory and specific arrest points (vessel segment and diameter) of the particles.

Model considerations and limitations: Conventional microfluidic channels are typically rigid and therefore do not fully recapitulate key features of pulmonary distal arterioles, including wall compliance, active vasoreactivity, and dynamic diameter regulation. To better approximate *in vivo* conditions, complementary designs may incorporate compliant materials, endothelialization, and tunable external pressure/strain to mimic vasomotion. Accordingly, microfluidic results should be interpreted as evidence for geometric size-based filtration under controlled conditions, while *ex vivo* perfused lung preparations and *in vivo* imaging remain necessary to validate arrest behavior in a compliant, actively regulated microcirculation.

Expected support: Single cells will largely pass the 20-40 µm channels and arrest only at the 5 µm channels; clusters 30 µm will preferentially lodge and cause complete occlusion in the 20–40 µm channels.

#### 6.2.2 *Ex vivo* perfused lung system

Protocol: Utilize an isolated, physiologically perfused mouse or rat lung system. Perfuse the lung with a controlled medium containing fluorescently labeled OS CTC clusters. Manipulate the local hemodynamics by adding agents (e.g., PGI2 analogues) to induce subpleural vasodilation or vasoconstrictors to alter the effective diameter of the filtering arterioles.

Readout: Map the 3D distribution of arrested emboli under different hemodynamic conditions using a confocal microscope or light-sheet microscopy optimized for cleared tissues.

Expected support: Conditions leading to localized vasodilation/flow enhancement in the subpleural zone should increase the stability of arrest, while conditions that significantly increase shear stress may promote cluster fragmentation or dislodgement.

### 6.3 Clinical imaging and pathology correlation: validation in humans

#### 6.3.1 Radiologic spatial mapping in cohorts

Protocol: Conduct a retrospective analysis of high-resolution CT scans from a large cohort of OS patients with documented pulmonary metastases. Standardize the lung volume and use semi-automated image analysis to segment the lung into concentric zones (e.g., Zone 1: subpleural 1.5–2 cm; Zone 2: mid-parenchyma; Zone 3: perihilar).

Readout: Calculate the metastasis density index (number of lesions per unit volume) for each zone.

Expected Support: The metastasis density index will be statistically highest in Zone 1 (subpleural) compared to Zone 2 and Zone 3, providing quantitative human evidence for the positional specificity.

#### 6.3.2 Correlative histopathology and microvascular analysis

Protocol: Obtain resected OS lung metastases (e.g., VATS resections). Perform detailed histopathological sectioning and use dual-immunohistochemistry staining: one marker for the tumor cells (e.g., Vimentin, Osteocalcin) and one marker for the pre-capillary arterioles (e.g., a smooth muscle actin stain) and capillaries (e.g., CD31).

Readout: Use high-magnification microscopy to measure the shortest distance between the edge of the earliest identifiable microscopic tumor foci and the nearest pre-capillary arteriole wall.

Expected support: Microscopic OS foci will show a high and specific spatial association (short distance) with the walls of the terminal pre-capillary arterioles, directly linking the initial colonization site to the hypothesized mechanical filter.

## 7 Discussion and implications

The Hemodynamic Mechanical-Filtration Hypothesis offers a spatially refined interpretation of the earliest stage of OS pulmonary metastasis. By moving the site of mechanical arrest from the universally distributed capillary bed to the anatomically restricted terminal arteriole, we provide a unified explanation for the long-observed subpleural bias.

### 7.1 Reframing metastasis theory: from organotropism to a spatial template

This hypothesis is intended as a spatially resolved refinement of contemporary mechanical perspectives rather than a rejection of Paget- or PMN-based concepts. Contemporary interpretations of Paget’s framework recognize that the “soil” is spatially heterogeneous within organs and that PMN priming, when present, precedes tumor-cell arrival through systemic tumor-derived factors.

In contrast, a distinct but related process unfolds after arrest and extravasation, when local endothelial, stromal, and immune remodeling supports survival and outgrowth; here we refer to this as metastatic niche maturation. Conflating this post-arrest maturation with PMN priming risks obscuring the temporal structure of metastasis.

Within this clarified framework, we propose that microvascular geometry and hemodynamic filtration provide an initial “spatial template” for seeding, particularly when circulating entities are sufficiently large or mechanically constrained. This spatial template can then interact with (i) pre-existing regional microenvironmental heterogeneity (including potential subpleural-specific tissue properties) and (ii) any upstream PMN priming, to determine whether an arrested entity progresses to a clinically detectable metastasis. Thus, the key contribution of the model is not that “soil is uniform,” but that anatomy- and flow-imposed constraints may be necessary to explain a reproducible subpleural pattern across patients.

### 7.2 Clinical relevance and translational opportunities

In this context, CTC–platelet aggregation may be viewed as a biomechanical amplifier of the proposed filtration process. By increasing embolic cohesion, shielding tumor cells, and favoring fibrin-rich microthrombus formation, platelets may shift a subset of circulating osteosarcoma material into a size/deformability range more likely to arrest at distal pulmonary arteriolar bottlenecks. If such bottlenecks are overrepresented within a defined subpleural distance, platelet-assisted embolic behavior would provide a plausible mechanistic bridge between intravascular aggregation and the clinically observed subpleural predominance (11).

The proposed framework is hypothesis-generating and its translational relevance should be framed as contingent on experimental validation. At present, the primary clinical value is to motivate measurable, spatially explicit endpoints (e.g., distance-to-pleura distributions of early foci) and to guide the design of studies that can discriminate between capillary-level versus distal-arteriolar filtration loci.

#### 7.2.1 Implications for imaging and risk stratification (near-term)

If distal filtration proves to be a dominant early constraint, radiologic mapping approaches that quantify lesion density by pleural distance may serve as useful research endpoints for cohort studies and for evaluating whether systemic interventions (e.g., anti-aggregation strategies) are associated with shifts in spatial patterns.

#### 7.2.2 Mechanistically aligned intervention concepts (long-term)

Several intervention ideas are conceptually consistent with the hypothesis, such as reducing cluster/aggregate integrity or modulating thrombosis-related processes, but these concepts are not unique to this manuscript and should be presented as long-term possibilities rather than immediate practice-changing recommendations. If future work confirms that embolic size/deformability causally determines arrest location, then anti-aggregation or anti-thrombotic strategies could be re-evaluated in osteosarcoma specifically, with spatial pattern endpoints informing mechanism-based trial design.

### 7.3 Generalizability and comparative oncology

The mechanical filtration model is potentially generalizable because it depends on physical parameters (effective embolus size and deformability) rather than tumor genotype alone. It predicts that tumor types more prone to circulating as cohesive clusters/aggregates may exhibit stronger distal or subpleural bias than those disseminating predominantly as small, highly deformable single cells. Comparative radiologic and pathological studies across tumor types could test whether embolic biomechanical profiles correlate with reproducible intra-lung spatial distributions.

Ultimately, this hypothesis shifts the research focus from solely molecular signaling to the mechanobiology of metastasis. It underscores that the metastatic process is not just a biological battle but a physical challenge where vascular geometry acts as an important early physical constraint.

## 8 Conclusion

The persistent subpleural predominance of osteosarcoma pulmonary metastases is a clinically robust but mechanistically underexplained spatial signature. The proposed Hemodynamic Mechanical-Filtration Hypothesis integrates contemporary biomechanics with refined mechanical perspectives to suggest that microvascular geometry and hemodynamic forces can bias the initial sites of embolic arrest, thereby establishing a spatial template for seeding. Subsequent progression depends on post-arrest metastatic niche maturation and may be facilitated or modulated by any upstream pre-metastatic niche priming, which by definition occurs prior to tumor cell arrival. If validated through the experimental strategies outlined, this framework would sharpen the mechanistic foundation of early lung seeding and motivate prevention concepts focused on embolic integrity and distal pulmonary microcirculatory conditions.

## 9 Limitations

Several limitations should be acknowledged when interpreting the Hemodynamic Mechanical-Filtration Hypothesis. First, current imaging and measurement technologies do not allow real-time, *in vivo* visualization of microscale hemodynamics at the level of terminal pre-alveolar arterioles (20–40 μm) in humans; thus, the earliest arrest events must be inferred from anatomical principles and model systems. Second, many *in vitro* microfluidic platforms employ rigid channels and therefore do not fully capture arteriolar compliance, vasoreactivity, and dynamic diameter regulation; microfluidic results should be interpreted as controlled tests of geometric filtration rather than complete replicas of the living pulmonary microcirculation. Third, the model does not explicitly incorporate the complex rheological behavior of red blood cells (RBCs), including deformability, axial migration, and non-Newtonian effects, which can influence shear profiles and effective luminal diameter in small vessels. Fourth, gravity-dependent perfusion gradients introduce regional variability in flow and vessel recruitment that may interact with (but not necessarily override) distal anatomical constraints. Together, these limitations reflect current technological constraints and delineate clear opportunities for future validation through advanced intravital imaging, RBC-integrated microfluidics, computational perfusion modeling, and physiologically perfused *ex vivo* lung systems. Fifth, although CTC–platelet aggregation and microthrombus formation are increasingly supported across multiple tumor types, direct osteosarcoma-specific evidence linking these processes to subpleural arrest remains limited and should be established experimentally.

## Data Availability

The original contributions presented in the study are included in the article/supplementary material. Further inquiries can be directed to the corresponding author.

## References

[1] Ward MP Kane LE Norris A Mohamed BM Kelly T Bates M . Platelets, immune cells and the coagulation cascade; friend or foe of the circulating tumour cell? Mol Cancer. (2021) 20:59. doi: 10.1186/s12943-021-01347-1. PMID: 33789677 PMC8011144

[2] Kempf-Bielack B Bielack SS Jürgens H Branscheid D Berdel WE Exner GU . Osteosarcoma relapse after combined modality therapy: an analysis of unselected patients in the cooperative osteosarcoma study group (COSS). J Clin Oncol. (2005) 23:559–68. doi: 10.1200/JCO.2005.04.063. PMID: 15659502

[3] Paget S . The distribution of secondary growths in cancer of the breast. 1889. Cancer Metastasis Rev. (1989) 8:98–101. doi: 10.1016/s0140-6736(00)49915-0, PMID: 2673568

[4] Ewing J . Neoplastic diseases: A treatise on tumors. W.B. Saunders (1928) p. 1–1134.

[5] Langley RR Fidler IJ . The seed and soil hypothesis revisited--the role of tumor-stroma interactions in metastasis to different organs. Int J Cancer. (2011) 128:2527–35. doi: 10.1002/ijc.26031. PMID: 21365651 PMC3075088

[6] Kaplan RN Riba RD Zacharoulis S Bramley AH Vincent L Costa C . VEGFR1-positive haematopoietic bone marrow progenitors initiate the pre-metastatic niche. Nature. (2005) 438:820–7. doi: 10.1038/nature04186. PMID: 16341007 PMC2945882

[7] Deng C Xu Y Chen H Zhu X Huang L Chen Z . Extracellular-vesicle-packaged S100A11 from osteosarcoma cells mediates lung premetastatic niche formation by recruiting gMDSCs. Cell Rep. (2024) 43:113751. doi: 10.1016/j.celrep.2024.113751. PMID: 38341855

[8] Albuquerque M Silva J Marchiori E Brandão Amorim V Menna Barreto M . CT features of osteosarcoma lung metastasis: A retrospective study of 127 patients. J Bras Pneumol. (2023):e20220433. doi: 10.36416/1806-3756/e20220433. PMID: 37132704 PMC10171270

[9] Ahmed G Elshafiey M Romeih M Kamel A Elgammal A Salama A . The prognostic value of the central location of pulmonary nodules in osteosarcoma patients. Discov Oncol. (2025) 16:705. doi: 10.1007/s12672-025-02446-x. PMID: 40343570 PMC12064495

[10] West JB . Fragility of pulmonary capillaries. J Appl Physiol (1985). (2013) 115:1–15. doi: 10.1152/japplphysiol.00229.2013. PMID: 23640584

[11] Pereira-Veiga T Schneegans S Pantel K Wikman H . Circulating tumor cell-blood cell crosstalk: Biology and clinical relevance. Cell Rep. (2022) 40:111298. doi: 10.1016/j.celrep.2022.111298. PMID: 36044866

[12] Sun Y Li T Ding L Wang J Chen C Liu T . Platelet-mediated circulating tumor cell evasion from natural killer cell killing through immune checkpoint CD155-TIGIT. Hepatology. (2025) 81:791–807. doi: 10.1097/HEP.0000000000000934. PMID: 38779918

[13] Gautam D Clarke EM Roweth HG Smith MR Battinelli EM . Platelets and circulating (tumor) cells: Partners in promoting metastatic cancer. Curr Opin Hematol. (2025) 32:52–60. doi: 10.1097/MOH.0000000000000852. PMID: 39508182

[14] Yang J Xu P Zhang G Wang D Ye B Wu L . Advances and potentials in platelet-circulating tumor cell crosstalk. Am J Cancer Res. (2025) 15:407–25. doi: 10.62347/JAYK5667. PMID: 40084364 PMC11897628

[15] Krog BL Henry MD . Biomechanics of the circulating tumor cell microenvironment. Adv Exp Med Biol. (2018) 1092:209–33. doi: 10.1007/978-3-319-95294-9_11. PMID: 30368755 PMC7304329

[16] Weiss L . Metastatic inefficiency. Adv Cancer Res. (1990) 54:159–211. doi: 10.1016/s0065-230x(08)60811-8. PMID: 1688681

[17] Aceto N Bardia A Miyamoto DT Donaldson MC Wittner BS Spencer JA . Circulating tumor cell clusters are oligoclonal precursors of breast cancer metastasis. Cell. (2014) 158:1110–22. doi: 10.1016/j.cell.2014.07.013. PMID: 25171411 PMC4149753

[18] Hagan CE Sheehan VM Rezanka CM Li MA Martínez-Escardó L Campbell KJ . Apoptotic cells promote circulating tumor cell survival and metastasis. Commun Biol. (2025) 8:1121. doi: 10.1038/s42003-025-08541-7. PMID: 40730678 PMC12307979

[19] Schulz A Loreth B Battmann A Knoblauch B Stahl U Pollex U . bone matrix production in osteosarcoma. Verh Dtsch Ges Pathol. (1998) 82:144–53. 10095426

[20] Cui J Dean D Hornicek FJ Chen Z Duan Z . The role of extracelluar matrix in osteosarcoma progression and metastasis. J Exp Clin Cancer Res. (2020) 39:178. doi: 10.1186/s13046-020-01685-w. PMID: 32887645 PMC7650219

[21] Townsley MI . Structure and composition of pulmonary arteries, capillaries and veins. Compr Physiol. (2012) 2:675–709. doi: 10.1002/cphy.c100081. PMID: 23606929 PMC3630377

[22] Kheyfets VO Rios L Smith T Schroeder T Mueller J Murali S . Patient-specific computational modeling of blood flow in the pulmonary arterial circulation. Comput Methods Programs BioMed. (2015) 120:88–101. doi: 10.1016/j.cmpb.2015.04.005. PMID: 25975872 PMC4441565

[23] Tang BT Pickard SS Chan FP Tsao PS Taylor CA Feinstein JA . Wall shear stress is decreased in the pulmonary arteries of patients with pulmonary arterial hypertension: An image-based, computational fluid dynamics study. Pulm Circ. (2012) 2:470–6. doi: 10.4103/2045-8932.105035. PMID: 23372931 PMC3555417

[24] Chu J Xiao LL Lin CS Liu S Zhang KX Wei P . Simulation of non-newtonian blood flow in diverging bifurcated vessels. J Appl Fluid Mechanics. (2024) 17:1204–16. doi: 10.47176/jafm.17.6.2329. PMID: 36128292

